# Fast track triage for COVID-19 based on a population study: The soda score

**DOI:** 10.1016/j.pmedr.2020.101298

**Published:** 2020-12-29

**Authors:** Javier Lopez-Pais, Diego López Otero, Teba Gonzalez Ferreiro, Carla Eugenia Cacho Antonio, Pablo José Antúnez Muiños, Marta Perez-Poza, Óscar Otero García, Victor Jimenez Ramos, Manuela Sestayo Fernández, María Bastos Fernandez, Xoan Carlos Sanmartin Pena, Alfonso Varela Roman, Manuel Portela Romero, Ana López Lago, Julián Álvarez Escudero, Alberto San Román, Jose Ramón Gonzalez-Juanatey, On behalf of CARDIOVID investigators

**Affiliations:** a Cardiology Department, University Clinical Hospital of Santiago de Compostela, Santiago de Compostela, Spain; b CIBERCV, Spain; c Instituto de Investigación Sanitaria IDICHUS, Spain; d Primary Healthcare, Centro de Salud Concepción Arenal, Santiago de Compostela, Spain; e Intensive Care Unit, University Clinical Hospital of Santiago de Compostela, Santiago de Compostela, Spain; f Anaesthesia Department, University Clinical Hospital of Santiago de Compostela, Santiago de Compostela, Spain; g Cardiology Department, University Clinical Hospital of Valladolid, Valladolid, Spain

**Keywords:** SARS, Severe Acute Respiratory Syndrome, SARS-COV-2, SARS Coronavirus 2, COVID-19, Coronavirus Disease 2019, ICU, Intensive Care Unit, IMV, Invasive Mechanical Ventilation, ROC, Receiver Operating Characteristic, SpO_2_, Peripheral O_2_ Saturation, Score, Triage, Fast-track, COVID-19, Prognosis

## Abstract

**Background:**

Healthcare systems are under prominent stress due to the COVID-19 pandemic. A fast and simple triage is mandatory to screen patients who will benefit from early hospitalization, from those that can be managed as outpatients. There is a lack of all-comers scores, and no score has been proposed for western-world population.

**Aims:**

To develop a fast-track risk score valid for every COVID-19 patient at diagnosis.

**Methods:**

Single-center, retrospective study based on all the inhabitants of a healthcare area. Logistic regression was used to identify simple and wide-available risk factors for adverse events (death, intensive care admission, invasive mechanical ventilation, bleeding > BARC3, acute renal injury, respiratory insufficiency, myocardial infarction, acute heart failure, pulmonary emboli, or stroke).

**Results:**

Of the total healthcare area population, 447.979 inhabitants, 965 patients (0.22%), were diagnosed with COVID-19. A total of 124 patients (12.85%) experienced adverse events. The novel SODA score (based on sex, peripheral O_2_ saturation, presence of diabetes, and age) demonstrated good accuracy for adverse events prediction (area under ROC curve 0.858, CI: 0.82–0.98). A cut-off value of ≤2 points identifies patients with low risk (positive predictive value [PPV] for absence of events: 98.9%) and a cut-off of ≥5 points, high-risk patients (PPV 58.8% for adverse events).

**Conclusions:**

This quick and easy score allows fast-track triage at the moment of diagnosis for COVID-19 using four simple variables: age, sex, SpO_2_, and diabetes. SODA score could improve preventive measures taken at diagnosis in high-risk patients and also relieve resources by identifying very low-risk patients.

## 1 Introduction

In December 2019, a cluster of severe acute respiratory syndrome (SARS) cases was first reported in Wuhan, the capital of the Chinese province of Hubei. A novel coronavirus was isolated and, after the viral genome was sequenced, it was named SARS-CoV-2 (Zhang et al., 2020, Zhang et al., 2020, Li et al., 2020, Huang et al., 2020). This new virus has a high contagion rate, with an R_0_ estimated of 3 (Riou and Althaus, 2020). By April of 2020, the disease caused by SARS-COV-2, known as COVID-19 (Coronavirus disease 2019), had spread over 200 countries, infecting almost two million people, and was labelled a global pandemic by the World Health Organisation (WHO) (World Health Organization, 2020). This situation threatens healthcare systems. To avoid their collapse, many countries have established exceptional measures, confining citizens in their homes and paralyzing economic activity, in order to buffer the spread of this disease (Anderson and Mckee, 2020).

Even so, the infection is mild or even asymptomatic in the majority of cases (Livingston and Bucher, 2020). One of the largest analyses conducted (Guan et al., 2020), reported more severe outcomes (intensive care [ICU] admission, mechanical ventilation, and death) in elderly male patients with hypertension, coronary artery disease, and diabetes (Chen et al., 2020). Most of the data available is based on observational studies developed in China, with a lack of information on how it affects the occidental population.

To avoid the collapse of healthcare systems, a fast and simple triage is mandatory to screen patients that will benefit from early hospitalization, from those who require close monitorization, and from those with low risk that can be managed as outpatients. There are scores to identify the risk of hospitalized COVID-19 patients with pneumonia (Ji et al., 2020), but the aim of this work is to generate a fast-track score valid for every COVID-19 patient at the moment of diagnosis.

## 2 Methods

### 2.1 Study design and participants

This single-centre, retrospective, observational study was performed at one University Hospital, which covers a population of 447.979 inhabitants who have been confined during the study. All laboratory-confirmed SARS-Cov-2 infected patients from the area, according to the interim guidance of the WHO (World, 2020), were included independent of their evolution. This study complied with the edicts of the 1975 Declaration of Helsinki and was approved by the institutional ethics of the institution. This paper this paper adheres to the TRIPOD guidelines as a framework for the development and reporting of a clinical risk prediction model.

### 2.2 Data collection

Standardized forms were used for the set-up of the database, including demographic information, epidemiological data, previous comorbidities and chronic treatments, and clinical data available at the moment the diagnostic test was performed (symptoms, fever and peripheral O_2_ saturation (SpO_2_). The data in source documents was confirmed independently by at least two physicians.

### 2.3 Outcomes

This work was conducted to develop an easy and fast score to assess the risk of adverse outcomes at the moment of COVID-19 diagnosis. The main outcome is a composite of major adverse events (death, ICU admission, invasive mechanical ventilation, bleeding > BARC3 (Kappetein et al., 2012), acute renal injury, respiratory insufficiency, myocardial infarction, acute heart failure, pulmonary emboli or stroke). As a secondary objective, the score will be validated on hospitalized patients and compared to available scores for COVID-19 in-hospital patients (the CALL score (Ji et al., 2020). The CALL score comprises four variables: comorbidity, age, lymphocyte count and lactate dehydrogenase at presentation.

### 2.4 Statistical analysis

Statistical analysis was performed with the IBM SPSS 24.0 software. Continuous variables are presented as means and standard deviations or as medians with standard deviation or interquartile range, as appropriate. Categorical variables are provided with percentages. Pearson chi-square or t-Student tests and their non-parametric equivalent were used depending on the variable type. Odds Ratios (OR) are reported with 95% confidence intervals. A two-sided P value of less than 0.05 was considered statistically significant.

The significance of each variable was assessed by a univariate and multivariate logistic regression model to investigate the independent high-risk factors of adverse events of illness. The immediate availability of the variables was mandatory for the correct implementation of the score. Only those that fulfilled that condition were included. All continuous variables with a statistically significant level on the univariate analysis were analyzed in order to identify optimal cut-off points. Variables with statistically significant level but with low prevalence in the population (<10%) were not included in the model. Logistic regression (backward stepwise method) was performed. The score was developed by the assignation of 1 point to each variable with OR lower than 4 and 2 points to those above. The performance of the novel score was assessed with receiver operating characteristic (ROC) curves. The area under the curve (AUC) and optimal cut-off values were determined and assessed by sensitivity, specificity and predictive values (PV).

## 3 Results

From March 10th to April 6th, 965 patients (0.22%) were diagnosed with COVID-19 out of the 447.979 inhabitants of the area covered by the university hospital. Table 1 summarizes age and sex distribution for COVID affection at a populational healthcare area level.

**Table 1:** Age and sex distribution of populational COVID-19 affection.

Age groups and sex (% of subgroup)	Population	Confirmed COVID-19 (% of pop.)	Hospitalization (% of cases)	ICU (% of cases)	Mortality (% of cases)	Adverse events (% of cases)
	N: 447.979	N: 965	N: 234	N: 33	N: 38	N: 124
**0**–**14 years**	**53.627**	**15 (0.03)**	**1 (6.67)**	**0 (0.00)**	**0 (0.00)**	**1 (0.00)**
Female		9	1 (6.67)	–	–	1 (6.67)
Male		6	0 (0.00)	–	–	–
**15**–**64 years**	**286.951**	**508 (0.18)**	**79 (15.55)**	**10 (1.97)**	**0 (0.00)**	**24 (4.72)**
Male		221	44 (19.9)	9 (4.07)	–	13 (5.88)
Female		287	35 (12.20)	2 (0.70)	–	11 (3.83)
**>64 years**	**107.401**	**442 (0.42)**	**154 (34.84)**	**23 (5.20)**	**38 (8.60)**	**99 (22.40)**
Male		198	93 (46.96)	15 (7.56)	30 (15.15)	69 (34.85)
Female		244	61 (25.42)	8 (3.33)	8 (3.33)	20 (8.20)

ICU: Intensive care unit. Adverse events (death, ICU admission, invasive mechanical ventilation, bleeding > BARC3, acute renal injury, respiratory insufficiency, myocardial infarction, acute heart failure, pulmonary emboli or stroke). BARC: Bleeding Academy Research Consortium. In the second block of columns the denominator is the total age subgroup population, in the third block of columns, the denominator is the total age and sex subgroup.

Out of the confirmed COVID-19 infected group, 124 patients (12.85%) experienced the main outcome, adverse events (a composite of death, ICU admission, invasive mechanical ventilation, bleeding > BARC3(13), acute renal injury, respiratory insufficiency, myocardial infarction, acute heart failure, pulmonary emboli or stroke). A total of 234 (24.25%) patients needed hospitalization, of those, 33 (14,1%) required ICU – 24 cases for IMV (9.8%)–. A total of 95 patients developed respiratory insufficiency (38.8%). During the period of the study, 38 patients died (3.94%), 35 developed acute heart failure (3.6%), 40 (16.3%) suffered AKI, 2 had bleedings worse than BACR3 (0.9%), 2 (0.9%) experienced myocardial infarction, and no pulmonary emboli or stroke was reported. Table 2 summarizes baseline characteristics of COVID-19 patients and a comparison between the group that experienced the outcome and those who did not.

**Table 2:** Baseline characteristics.

	Total Cohort (N:965)	No-Adverse Events (N:841)	Adverse Events (N:124)	P-Value
*Clinical presentation*				
Days with symptoms	6.1 ± 4.6	6.23 ± 4.79	5.96 ± 4.54	0.369
Fever (referred)	59.9	54 (78.3)	524 (59.1)	0.002
SaO2 < 95%	30.5	167 (25.5)	53 (77.9)	<0.001

*Demographic characteristics*				
Age, years	59.5 ± 20.3	58.24 ± 20.21	76.39 ± 11.58	<0.001
Male sex	43.9	21 (30.4)	520 (58)	<0.001
Health worker	13.1	1 (1.4)	125 (14.0)	0.003
*Pneumopathy*				
Pulmonary disease	11.9	16 (23.5)	99 (11.1)	0.002
COPD/Asthma	8.9	11 (17.5)	75 (9.2)	0.032
OSAHS	2.5	5 (7.9)	19 (2.3)	0.008

*Cardiovascular profile*				
Tobacco	10.3	15 (21.7)	84 (9.4)	0.001
Hypertension	30.9	34 (49.3)	264 (29.6)	0.001
Diabetes	12.8	32 (46.4)	92 (10.3)	<0.001
Dyslipidaemia	28.2	37 (53.6)	235 (26.3)	<0.001
CAD	4.4	11 (16.2)	31 (3.5)	<0.001
Depressed LVEF	1.6	6 (0.7)	9 (14.8)	<0.001
Stroke/TIA	3.1	25 (2.8)	5 (7.2)	0.041
Peripheral vasculopathy	2.7	12 (1.3)	14 (20.6)	<0.001

*Other comorbidity*				
GFR < 30 mL/min	3.0	20 (2.2)	9 (13.2)	<0.001
Active cancer	2.5	21 (2.4)	3 (4.4)	0.294
Autoimmune disease	2.9	23 (2.6)	5 (7.4)	0.024

Values are mean ± standard difference or n (%).

COPD: Chronic Obstructive Pulmonary Disease. CAD: Coronary artery disease. LVEF: Left ventricle ejection fraction. OSAHS: Obstructive Sleep Apnea-Hypopnea Syndrome. SaO2: Arterial Oxygen Saturation. TIA: Transient Ischemic Attack. GRF: Glomerular filtration rate.

Table 3 details multivariate analysis based on logistic regression and development of the score. The analysis shows that male sex (OR: 2.39, CI 1.49–3.84), age (more than 50 years old: OR: 2.6, CI: 1.01–6.77; and more than 70 years old: OR: 5.2, CI 2.08–13.22), SpO_2_ lower than 95% (OR: 7.33, CI 4.45–12.08), and presence of diabetes (OR: 3.19, CI 1.87–5.43) were independent high-risk factors for adverse outcomes. To facilitate clinical implementation of these predictors, a novel score model was created, named SODA.

**Table 3:** Multivariate analysis for SODA score.

	OR	CI	P-Value	SODA score punctuation
SaO2 < 95%	7.33	4.45–12.08	<0.001	2
More than 50 years old	2.6	1.01–6.77	0.048	1
More than 70 years old	5.2	2.08–13.22	<0.001	2
Male sex	2.39	1.49–3.84	<0.001	1
Diabetes	3.19	1.87–5.43	<0.001	1

OR: Odds Ratio. CI: 95% Confidence Interval.

SaO2: Arterial Oxygen Saturation.

**Fig. 1:**
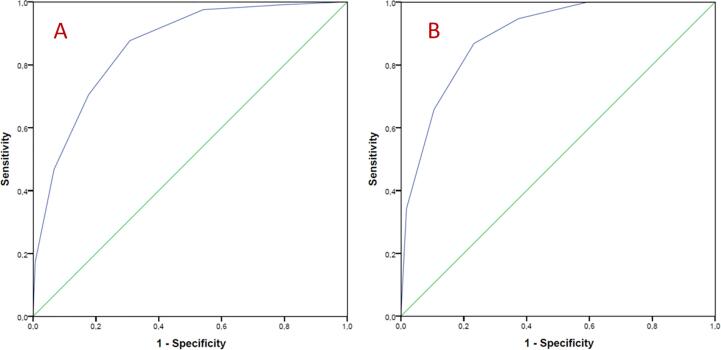
Receiver Operating Characteristic curve of SODA score to predict: A, adverse events (death, ICU admission, invasive mechanical ventilation, bleeding > BARC3, acute renal injury, respiratory insufficiency, myocardial infarction, acute heart failure, pulmonary emboli or stroke). Area under curve 0.858, CI: 0.82–0.98, p < 0.001. B, mortality. Area under curve 0.89, CI: 0.58–0.94, p < 0.001.

**Fig. 2:**
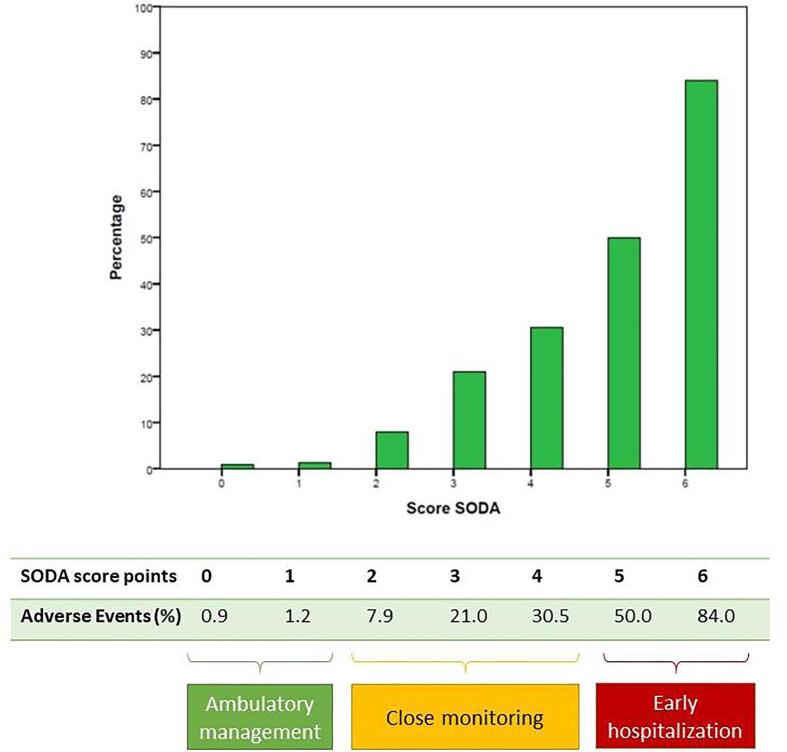
Percentage of adverse events in each punctuation of the SODA score (green) and percentage of patients without adverse events (blue). Adverse events: death, ICU admission, invasive mechanical ventilation, bleeding > BARC3, acute renal injury, respiratory insufficiency, myocardial infarction, acute heart failure, pulmonary emboli, or stroke. (For interpretation of the references to colour in this figure legend, the reader is referred to the web version of this article.)

The performance of the novel score was assessed with a ROC curve. SODA score demonstrated good accuracy for adverse events prediction (AUC 0.858, CI: 0.82–0.98, p < 0.001) Fig. 1. Two cut-off points were set. First one, SODA score 0 or 1 point, identifies patients with low risk of adverse events (positive PV for absence of events: 98.9%). Second cut-off point, was established to identify high-risk patients and was set on 5 or 6 points (positive PV 58.8% for adverse events), The SODA score also showed good accuracy when taking only mortality into account (AUC 0.89, CI: 0.58–0.94, p < 0.001). Table 4 explains the accuracy of the model for estimating risk of adverse events. Fig. 2 reflects the percentage of adverse events in each punctuation of the SODA score.

**Table 4:** Accuracy of the SODA model for estimating the risk of adverse events (death, ICU admission, invasive mechanical ventilation, bleeding > BARC3, acute renal injury, respiratory insufficiency, myocardial infarction, acute heart failure, pulmonary emboli or stroke) based on the 965 COVID patients enrolled.

Variable	Value (%)
**Cut-off**	**<=1**
Sensitivity	45.8
Specificitiy	97.5
Positive Predictive Value	98.9
Negative Predictive Value	1.1
**Cut-off**	**>=5**
Sensitivity	46.7
Specificitiy	93.3
Positive Predictive Value	58.8
Negative Predictive Value	89.6

BARC: Bleeding Academy Research Consortium.

**Fig. 3:**
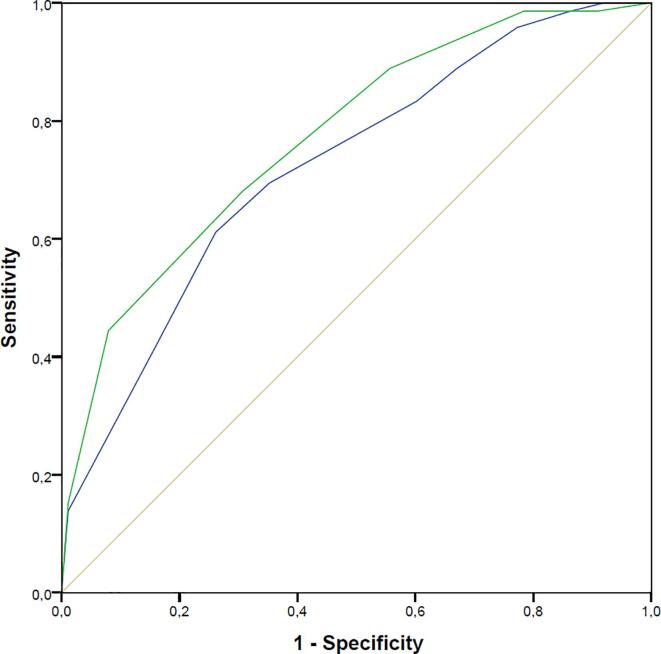
Evaluation of SODA score in hospitalized patients and comparison with CALL score, a specific score for hospitalized COVID-19 patients. Even in this subgroup of patients, SODA score (green) demonstrates good accuracy for adverse events, with a higher AUC (0.77, CI 0.70–0.83) when compared to the CALL score (blue): AUC 0.71, CI 0.63–0.79. (For interpretation of the references to colour in this figure legend, the reader is referred to the web version of this article.)

Although SODA is designed for all-comer COVID-19 patients at the moment of their diagnosis, as a secondary objective, it was evaluated in hospitalized patients and compared with the CALL score, a specific score for hospitalized COVID-19 patients. Fig. 3 shows that even in this subgroup of patients, SODA score demonstrates good accuracy for adverse events, even with a higher AUC (0.77, CI 0.70–0.83) when compared to CALL score (AUC 0.71, CI 0.63–0.79).

## 4 Discussion

This easy-to-use and fast-to-obtain score will allow fast-track triage at the moment of diagnosis for COVID-19 using four simple variables: sex, SpO_2_, diabetes, and age. Taking into account that the majority of the population are outpatients; this score may help clinicians to distinguish between patients at high risk of events who benefit from early hospitalization, from those who require close monitoring, and from those of low risk that can be managed as outpatients. It is important to highlight that this triage score, like any other, should not be considered in patients in an obvious critical situation, in which immediate active measures are mandatory.

In addition to its high accuracy, the main advantage of the SODA score herein described is its simplicity, with items that can be assessed immediately and precisely at the very moment the COVID-19 test is performed. Other models explored had better validation parameters, but included variables with less immediate and precise availability, a mandatory fact for the correct implementation of the score. SODA score can be calculated in less than a minute by answering three simple questions and by using a pulse oximeter.

Most healthcare systems have suffered significant stress or have even collapsed; tools like this score may be useful to avoid that happening again in a feasible second wave (Xu and Li, 2020). However, if the pandemic is putting healthcare systems of wealthy countries to the test and pushing them to the limit, in all likelihood, it will be devastating when the outbreak hits developing countries with inadequate healthcare systems. The SODA score will surely help healthcare workers decide whether or not a patient can be sent home safely. Public policies could implement this score to lighten hospital burden.

The interaction between SARS-COV-2 and alveoli, mainly through ACE 2, triggers a massive production of proinflammatory cytokines which attract leucocytes and hyper-active macrophages which liberate more cytokines, inducing the obliteration of the alveoli and the development of characteristic hyaline membranes of the acute respiratory distress syndrome (ARDS) (Zhang et al., 2020, Zhou et al., 2020, Yan et al., 2020). Its high inflammatory load can induce myocarditis and arrhythmias, and may generate pro-thrombotic status, which favours acute coronary syndromes (Guo et al., 2020, Zhang et al., 2020, Chapman et al., 2020). Once the cascade of immune response is initiated, patient deterioration occurs fast, therefore preventive measures are mandatory.

Age, hypertension, lymphopenia, D-dimer, cardiac troponin, interleukin-6 or ferritin have been proposed as markers of poor prognosis (Guo et al., 2020, Wang et al., 2020, Yang et al., 2020, Zhou et al., 2020). Aside from significant laboratory abnormalities, chest X-ray and computed tomography findings were also related to the evolution of the disease (Guan et al., 2020). Traditional scores used in critical patients, like the SOFA score, have shown good discrimination power in helping physicians identify patients with poor prognosis at an early stage (Zhou et al., 2020). Recently, the CALL score (Ji et al., 2020), a specific score for in-hospital COVID-19 patients, demonstrated high accuracy predicting the progression of patients with pneumonia. Of note, this score needs a blood sample analysis to be performed. However, the SODA score only needs three easy questions to be answered and a pulse oximeter.

The score proposed in this work has three main strengths when compared to the remaining scores available. First, it is specific for the initial stages of the disease and valid both for hospitalised and ambulatory patients. Second, its extraordinary simplicity and the wide availability of the items required, position the score as a useful fast-track triage tool. Third, being developed based on a whole-population study, it avoids the possible bias of other scores developed on a specific subgroup of COVID-19 patients.

## 5 Study limitations

Because of its observational nature, unmeasured confounders could constrain causal inference in the present study. Including every single COVID-19 patient of the area mitigates this fact. Despite the good performance of our proposed SODA score for risk stratification in the population of this study, external validation is needed in a different cohort of patients.

## 6 Conclusions

This quick and easy score allows fast-track triage at the moment of COVID-19 diagnosis using four simple variables (sex, SpO_2_, diabetes, and age) and is useful for ambulatory as well as for hospitalized patients. This score clearly identifies patients at high risk of developing adverse outcomes and those at very low risk. SODA score could improve preventive measures taken at the moment of diagnosis in high risk patients and also relieve resources with the identification of very low risk patients.

Most healthcare systems have suffered significant stress or have even collapsed during the pandemic; tools like this score will surely avoid that happening again in a feasible second wave or in developing counties.

## Authorship statement

JLP and DLO contributed to the conception or design of the work and final approval of the version to be published. TBG, CECC, PJAM, MPP, OOG, VJR, MBF, XCSP, AVR and MPR contributed to the data collection. JLP, DLO, TGF, CECC, and PJAM contributed to the data analysis and interpretation. JLP and DLO drafted the article. AVR, MPR, ALL, APR, JAE, ASR and JRGJ contributed to the critical revision of the article.

## Fundings

I, the corresponding author, have full access to all the data in the study and have final responsibility for the decision to submit for publication.

The authors received no financial support for the research, authorship, and/or publication of this article.

## Declaration of Competing Interest

The authors declare that they have no known competing financial interests or personal relationships that could have appeared to influence the work reported in this paper.
